# Supplementary material for: Poly(ADP-ribose)-binding protein RCD1 is a plant PARylation reader regulated by Photoregulatory Protein Kinases
Source: Commun Biol. 2023 Apr 19;6:429. doi: 10.1038/s42003-023-04794-2 (PMC10115779; doi:10.1038/s42003-023-04794-2)
Supplement: Supplementary file 2 — Supplementary Information [file 42003_2023_4794_MOESM2_ESM.pdf]

Supplementary table 1.1. Identified RCD1 phosphosites

Peptide sequence	Contained phosphosites	Study
VLDssRCEDGFGK	S11, S12	*in vitro* PPK (a)
AAsYAAYVtGVsCAK	S27, T33, S36	*in vivo* (b), *in vitro* PPK (a)
LEIDVNGGEtPR	T204	*in vivo* (a, b)
LNLEECsDEsGDNMMDDVPLAQR	S213, S216	*in vitro* PPK (a)
ssNEHYDEAtEDCsR	S230, S231, T239, S242, S244	*in vivo* (b), *in vitro* PPK (a)
KLEAAVsK	S252	*in vivo* (a), *in vitro* PPK (a)
WDEtDAIVVsGAK	T257, S263	*in vivo* (b)
LTGsEVLDK	S270	*in vitro* PPK (a)
FssEIAEAR	S301, S302	*in vitro* PPK (a)
QVEItKK	T319	*in vitro* PPK (a)
DNsGVtLEGPK	S467, T470	*in vivo* (a), *in vitro* PPK (a)
GsGsANsVGssttRPK	S490, S492, S495, S498, S499, T500, T501	*in vivo* (a), *in vitro* PPK (a)
EIPGsIR	S578	*in vitro* PPK (a)

(a) this study, (b) Wirthmueller *et al*, 2018.
Phosphopeptides between the WWE and PARP-like domains are marked with *italic*.
Lowercase s and t represent phosphorylated serine and threonine residues respectively.

Supplementary table 1.2. Identified RCD1 phosphopeptides

Peptide sequence	Best Site Probabilities	Mascot Ion Score	Replicates
VLDsSRcEDGFGK	99,69	48	2
VLDSsRcEDGFGK	99,69	48	2
AAsYAAYVTGVScAK	100	76	2
AASYAAYVTGVscAK	100	71	2
AAsYAAYVTGVscAK	100; 100	48	2
LEIDVNGGEtPR	100	31	2
LNLEEcsDEsGDNmmDDVPLAQR	100, 100	60	2
KLEAAVsK	100	53	3
LTGsEVLDK	100	52	3
FsSEIAEAR	100	53	2
FSsEIAEAR	100	71	2
QVEItKK	100	33	2
DNsGVtLEGPK	100; 100	61	2
DNsGVTLEGPK	100	65	2
DNSGVtLEGPK	100	54	2
GSGSANsVGsSTTRPK	100; 99.47	75	5
GSGsANSVGSSTTRPK	98,53	65	4
GSGSANsVGSSTTRPK	98,53	65	4
GSGSANSVGsSTTRPK	99,45	58	2
GsGSANSVGSSTtRPK	98.46; 98.46	44	2

GsGSANSVGSSttRPK	99.99; 98.64; 99.99	42	2
GSGSANSVGSsttRPK	99.99; 98.64; 99.99	41	2
EIPGsIR	100	31	3

Lowercase s and t represents phosphorylated serine and threonine.
lowercase c represents carbamidomethylated cysteine, lowercase m represents oxidized methionine

Supplementary table 2. Primers used in the study

RCD1 *nls1/2*

RCD1 NLS mutations were generated on the basis of pGWB13:RCD1(promoter)_RCD1(genomic sequence)_3xHA, and on the basis of pBm43GW:UB10(promoter)_RCD1(CDS)_3xVenus.

The function of NLS1 and NLS2 was perturbed by mutating the RCD1 genomic sequence using the following primers. Mutagenesis of NLS1 and of NLS2 was performed sequentially.

nls1_F:	GTGAAGATGGATTCGGAAAActGAtcAAGCGCGCAGCAAGCTATGC
nls1_R:	GCATAGCTTGCTGCGCGCTTgaTCagTTTTCCGAATCCATCTTCAC
nls2_F:	GTGCCAAATTCCTGACAAAAtcAtcAGGCTGGAAGGTGAAAACAA
nls2_R:	TTGTTTTCACCTTCCAGCCTgaTgaTTTTGTCAGGAATTTGGCAC

RCD1-Venus [pBm43GW:UB10(promoter)_RCD1(CDS)_3xVenus]

RCD1-Venus construct [pBm43GW:UB10(promoter)_RCD1(CDS)_3xVenus] was assembled by multiple Gateway reaction in the destination vector pBm43GW.

The individual entry vectors were generated using the following primers.

UBIQUITIN10 promoter

attB4F-pUB10:	GGGGACAACTTTGTATAGAAAAGTTGAAgtcgacgagtcagtaataaacg
attB1R-pUB10:	GGGGACTGCTTTTTTGTACAAACTTGTctgttaatcagaaaaactcagat

RCD1 CDS

attB1F-RCD1:	GGGGACAAGTTTGTACAAAAAAGCAGGCTaaATGGAAGCCAAGATCGTCA
attB2R-RCD1:	GGGGACCACTTTGTACAAGAAAGCTGGGTcCAATCCACCTGCACCTTCTT

RCD1 domain deletion constructs were generated on the basis of pGWB13:RCD1(promoter)_ _RCD1(genomic sequence)_3xHA, and on the basis of pBm43GW:UB10(promoter)_RCD1(CDS)_3xVenus.

The deletions were introduced by PCR followed by In-Fusion (Clontech). The following primers were used.

dWWE-R:	AGCGATTCCAGTCTTCTTAAAATAAGTG
dWWE-F:	GCATGGATTGACAATGCAGG
dPARP-R:	ACTGGAAAAACGGCCCACA
dPARP-F:	GAATTTGTTGTTAGGTTCAAGCTGT
dRST-F:	gACCCAGCTTTCTTGTACAA
dRST-R:	CAAGAAAGCTGGGTcAATCAAATTACCTTCAGCATTGG

RCD1 IDR2 swap was done using Type IIS restriction enzyme cloning amplifying the vector and insert with the following primers

IDR2-F:	ATGGTCTCCGAAGCCAAGATCGTCAAG
IDR2-R:	ATGGTCTCCTCCCAACACCATAGATGGACT
pBm43GW-F:	ATGGTCTCCGGGATCCATTTAACC GCTG
pBm43GW-R:	ATGGTCTCGCTTGACGATCTTGGCTTCC
pDONR-F:	ATGGTCTCCCTTCCATTTCTATATATTAACAATACTAAAC
pDONR-R:	ATGGTCTCCGGGATCCATTTAACCGCTG

RCD1 constructs used in RCD1-PAR interaction experiments

RCD1-His F	TTTTTTCTCGAGCATGGAAGCCAAGATCGTC
RCD1-His R	TTTTTTACGCGTTTACAATCCACCTGCACC
GSRT-RCD1 F	5'-GAATTCATGGAAGCCAAGATCGTCAA
GST-RCD1 R	5'-GCGGCCGCTTACAATCCACCTGCACCTTC
GST-WWE$_{RCD1}$ F	5'-GAATTCATGGAAGCCAAGATCGTCAAGGTG
GST-WWE$_{RCD1}$ R	5'-GCGGCCGCTTAGTCAATCGATGCAAGAGGGGTTTT
GST-RCD1ΔWWE F	5'-GAATTCATGGATAGCTGCAGCCGCAAGCTCGAA
GST-RCD1ΔWWE R	5'-GCGGCCGCTTACAATCCACCTGCACCTTCTTC

GST-RCD1ΔPARP-like F	Phos-GAATTTGTTGTTAGGTTCAAGCTGT
GST-RCD1ΔPARP-like R	Phos-ACTGGAAAAACGGCCCACA
GST-RCD1- $^{S/T}$IDR2 ^{A}-F:	TTCCAGGGGCCCCTGGGATCCGAAGCCAAGATCGT
GST-RCD1- $^{S/T}$IDR2 ^{A}-R:	CTGCAGCGGTTAAATGGATCCCAACACCATAGATG

PPK and RCD1 pENTR constructs, these were recombined in Gateway LR reactions with destination vectors pH7RWG2 (RFP), pGWB414 (3HA) or pK7FWG2 (GFP)

**PPKs**	
PPK1_pENTR/D-Topo_fw	CACCATGCCGGAGCTTCGCCG
PPK1_pENTR/D-Topo_rv	AGATACAGTTCGGCCATAGC
PPK2_pENTR4_fw (Gibson)	CAAAAAAGCAGGCTCCACATGCCAGAGTTAAGAAGTGG
PPK2_pENTR4_rv (Gibson)	GCTGGGTCTAGATATCTCGAGTT GCAAACTGTCCTCCCAAAG
PPK3_pENTR/D-Topo_fw	CACCATGCCAGAGTTAAGAAGTGG
PPK3_pENTR/D-Topo_rv	GCAAACTGTCCGACCATAG
PPK4_pENTR/D-Topo_fw	CACCATGCCTGAGCTGCGTAGC
PPK4_pENTR/D-Topo_rv	TGACACAGTTCGACCATAAC
**RCD1**	
RCD1_pENTR/D-Topo_fw	CACCATGGAAGCCAAGATCGTCAAG
RCD1_pENTR/D-Topo_rv	CAATCCACCTGCACCTTCTTC

GST-PPK

PPKs were cloned into a modified pGEX6-P1, in wich an AsiSI site was introduced into the MCS.
Cloning was done using the InFusion system with linker regions indicated in italic, conferring to the AsiSI restriction site

PPK1_cloneF	GGATCTGCTGGTGCG ATGCCGGAGCTTCGCCGTGGAGT
PPK1_cloneR	CCAGCGCTACCAGCGCC AGATACAGTTCGGCCATAGC
PPK2_cloneF	GGATCTGCTGGTGCG ATGCCAGAGTTAAGAAGTG
PPK2_cloneR	CCAGCGCTACCAGCGCC GCAAACTGTCCTCCCAAAGCATA
PPK3_cloneF	GGATCTGCTGGTGCG CCAGAGTTAAGAAGTGGAGCAAGGAGATC
PPK3_cloneR	CCAGCGCTACCAGCGCC GCAAACTGTCCGACCATAGCATAT
PPK4_cloneF	GGATCTGCTGGTGCG ATGCCTGAGCTGCGTAGCAA
PPK4_cloneR	CCAGCGCTACCAGCGCC ATGACACAGTTCGACCATAACAA
GST-RCD1_T204A	
T204A_F	TTAATGGTGGCGAGGCACCGAGGTTAAATTTG
T204A_R	CAAATTTAACCTCGGTGCCTCGCCACCATTAA

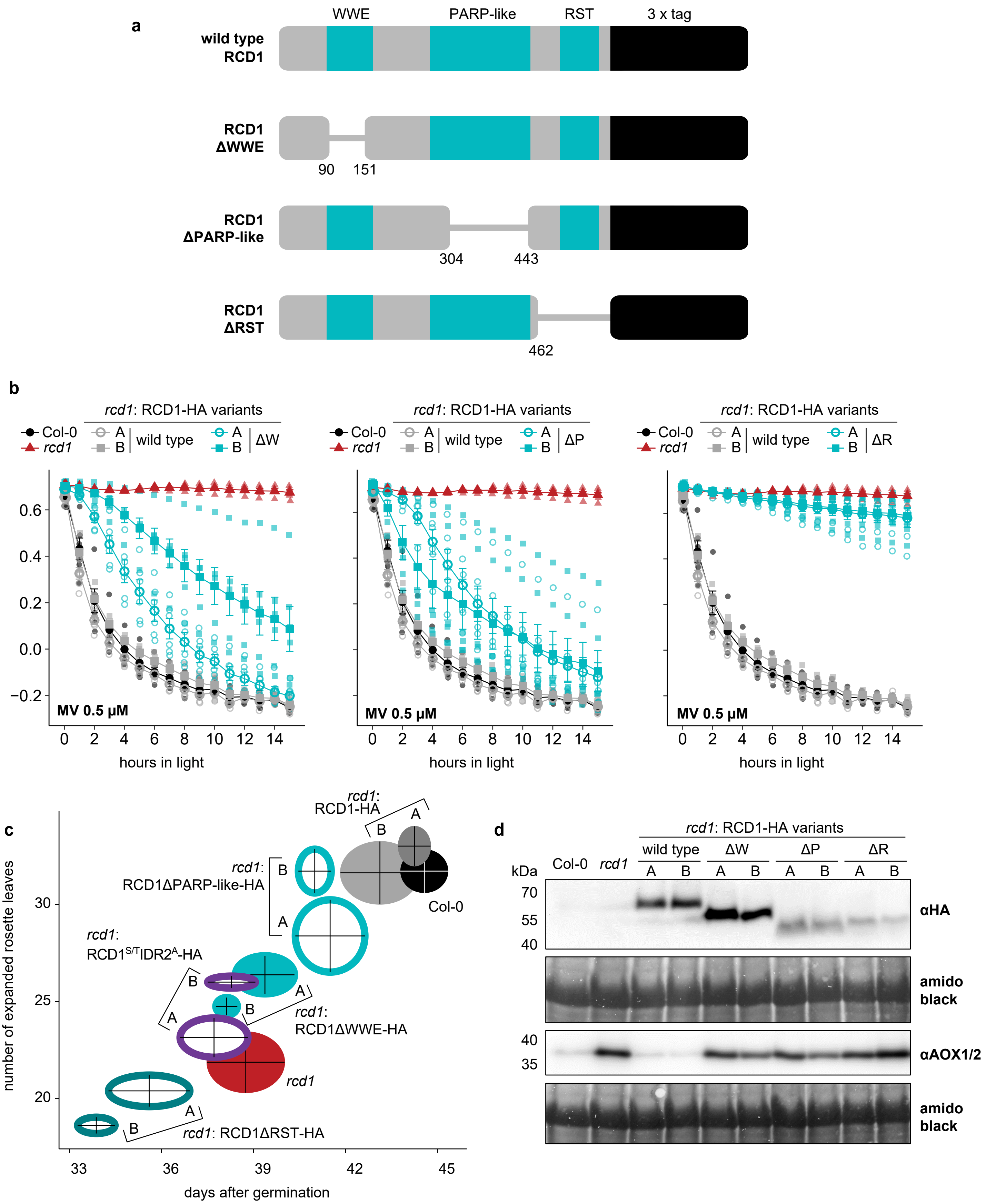

Supplementary figure 1

Supplementary figure 1. Characterization of RCD1 domain deletion mutants.

a. Schematic representation of RCD1 domain deletion constructs fused to triple HA or triple Venus tag and expressed in *rcd1* background.

b. MV sensitivity is not restored in lines expressing RCD1ΔWWE-HA (ΔW) and RCD1ΔPARP-HA (ΔP), and RCD1ΔRST-HA (ΔR). Two independent lines for each construct (A and B) were used in the experiments. PSII inhibition (Fv/Fm) by MV was measured in indicated lines using 0.5 μM MV. For each experiment, leaf discs from three individual rosettes were used. The experiment was performed three times with similar results. Source data and statistics are presented in **Supplementary Data 1**.

c. Early flowering time phenotype of *rcd1* is not reverted in lines expressing RCD1ΔWWE-HA (ΔW), RCD1ΔPARP-like-HA (ΔP), RCD1ΔRST-HA (ΔR), and RCD1$^{S/T}$IDR2^{A}-HA. Two independent lines for each construct (A and B) were used in the experiments. Flowering time defined as the day of the opening of the first flower after germination, is plotted against the number of expanded rosette leaves on the flowering day. The experiment was performed three times with similar results.
Source data and statistics are presented in **Supplementary Data 1**.

d. Immunoblot analysis of two independent domain deletion lines for each construct (A and B) shows presence of RCD1-HA in complementation lines (upper panel) and increased AOX1/2 expression in these lines at the level similar to the *rcd1* mutant (middle panel). Rubisco large subunit detected by amido black staining is shown as a control for equal protein loading.

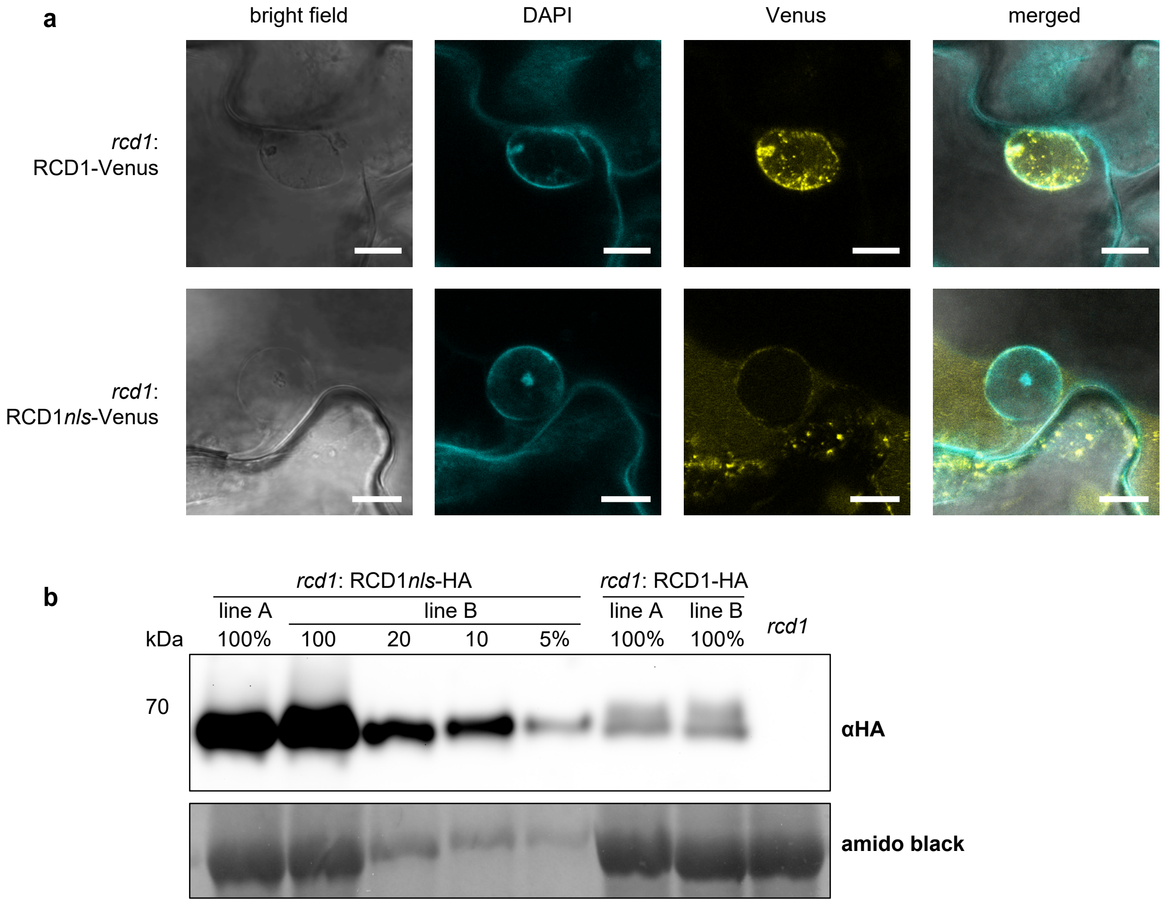

Supplementary figure 2. Nuclear localization of RCD1.

a. RCD1*nls*-Venus is localized outside the nuclei. Confocal images were taken from stable Arabidopsis lines expressing full-length RCD1-Venus and RCD1*nls*-Venus. DAPI staining was used to highlight nuclear structures. White bars indicate 10 µm.

b. Disruption of NLS leads to higher RCD1 accumulation in plants. Abundance of RCD1-HA in RCD1*nls*-HA and RCD1-HA lines was assessed by immunoblot analysis with HA-specific antibodies. A total protein amount of 100 µg corresponds to 100%. Rubisco large subunit detected by amido black staining is shown as a control for protein loading.

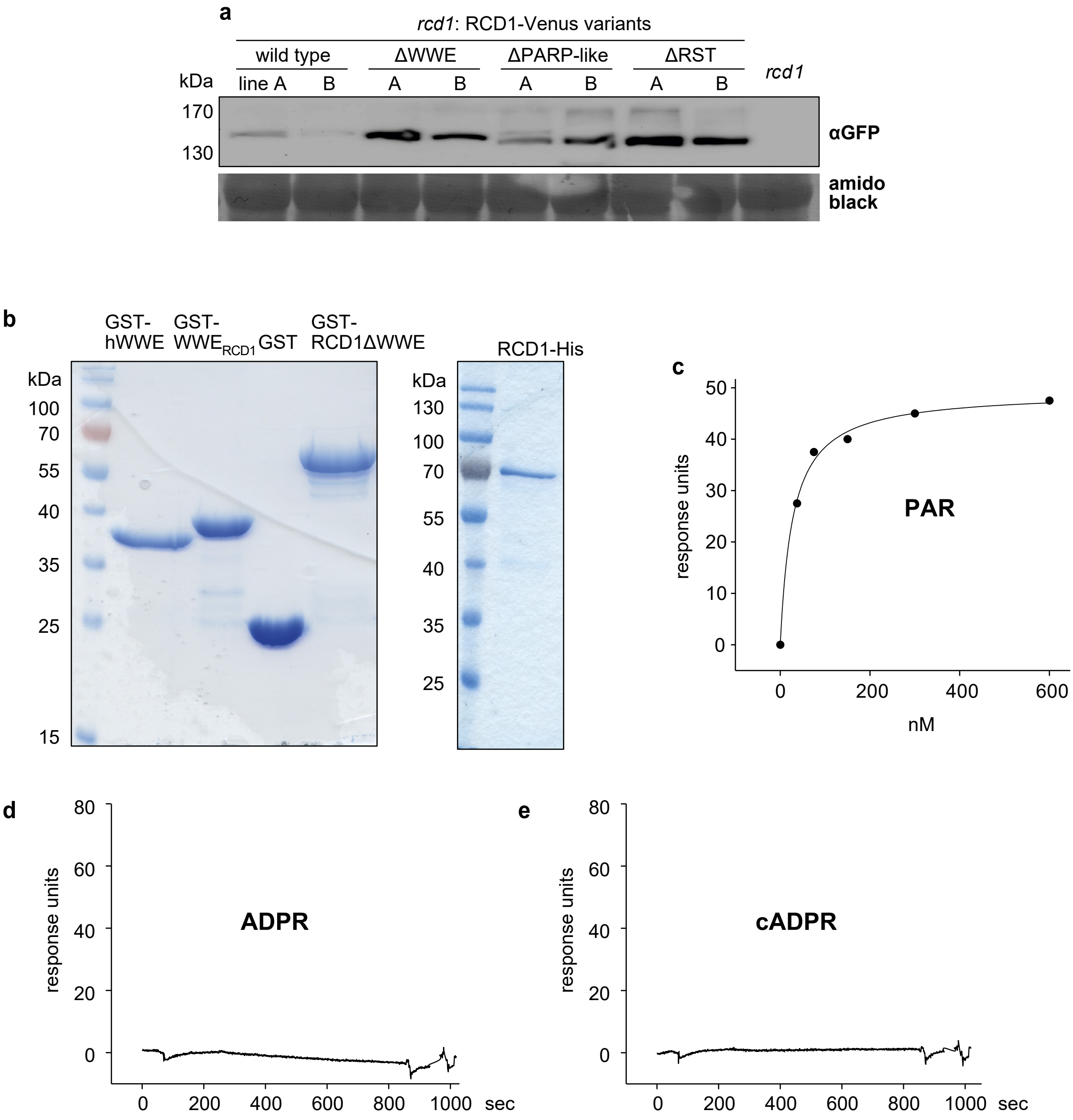

Supplementary figure 3. RCD1 binds PAR but not ADP-ribose or cyclic ADP-ribose.

a. Domain deletion does not lead to decreased abundance of RCD1. RCD1 level in indicated lines was assessed by immunoblot analysis of total protein extracts with GFP-specific antibody. A total amount of 100 µg protein was loaded per lane. Rubisco large subunit detected by amido black staining is shown as a control for protein loading.

b. The purity of recombinant proteins used in *in vitro* analyses of PAR binding. Proteins were purified, resolved by SDS-PAGE and stained with Coomassie.

c. PAR titration curve obtained by SPR analysis of PAR binding by RCD1-His. The curve was plotted using non-linear regression with the assumption of one-to-one binding.

d, e. RCD1-His binds neither mono-ADP-ribose (ADPR), nor cyclic ADP-ribose (cADPR). SPR sensorgrams do not show any response in case of ADPR or cADPR profiled at 1 mM concentrations over immobilized RCD1-His.

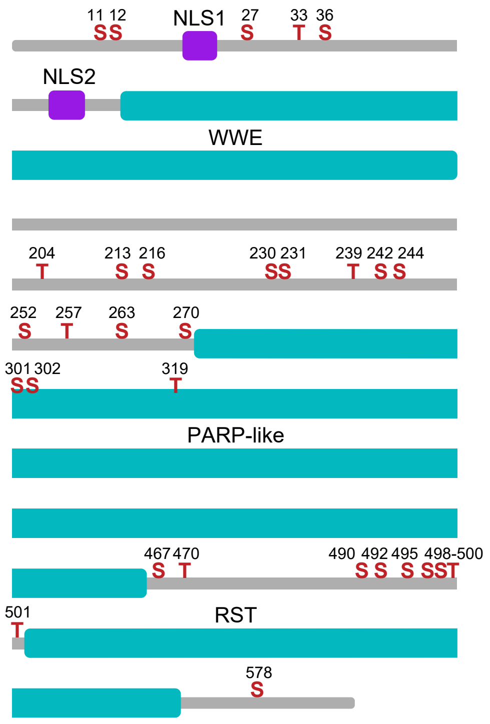

Supplementary figure 4. Schematic representation of RCD1 phosphosites.

RCD1 phosphosites identified by *in vivo* and *in vitro* analyses as described in **Supplementary table 1**. RCD1 domains are highlighted in blue and intrinsically disordered regions are indicated in grey. Individual phosphosites are marked in red and numbered.

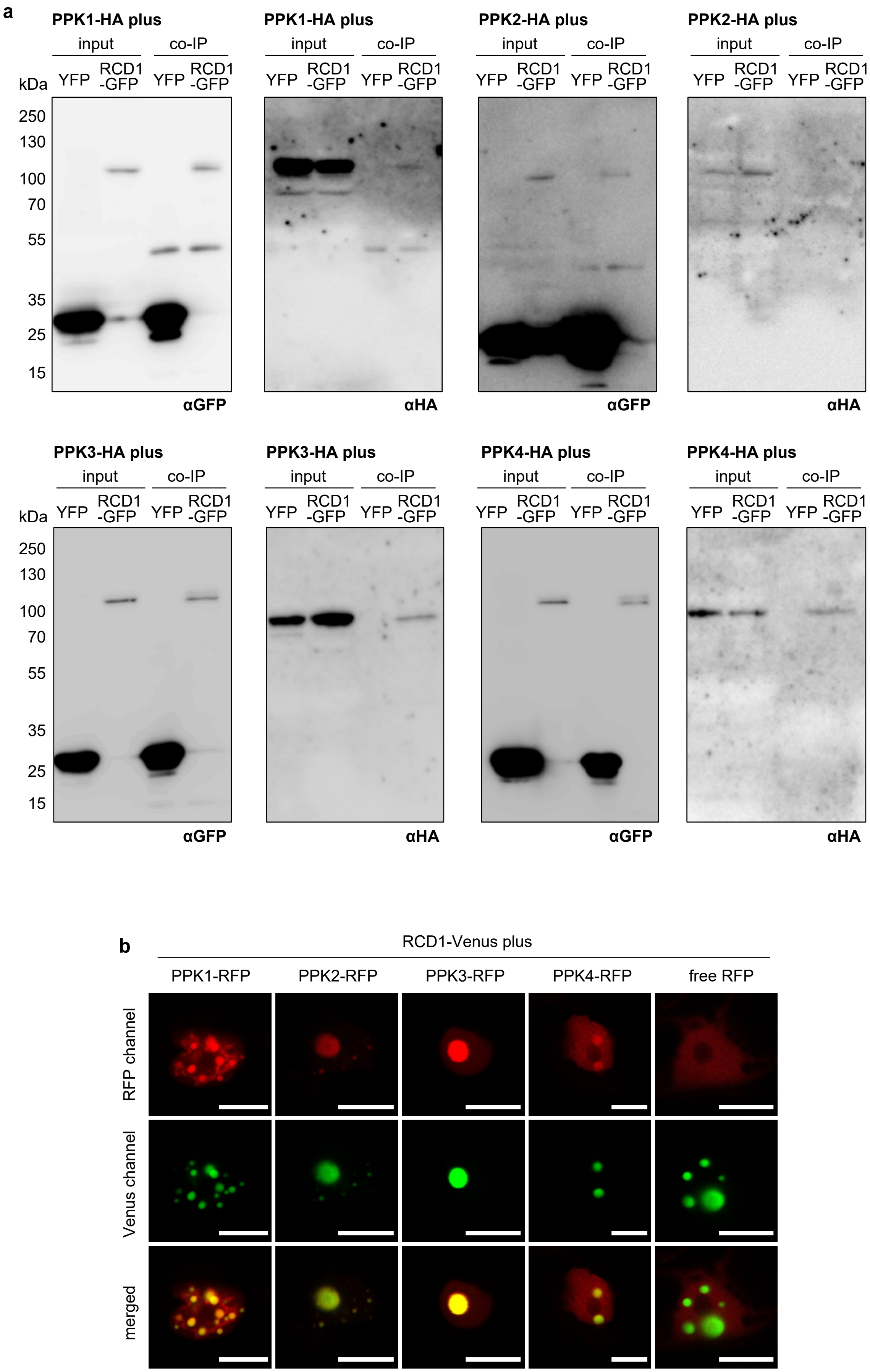

Supplementary figure 5.

Supplementary figure 5. RCD1 and PPKs interact *in vivo*.

a. RCD1-GFP interacts with PPK-HA in tobacco. RCD1-GFP was transiently co-expressed with HA-tagged versions of PPK1, 2, 3 or 4 in *N. benthamiana*. YFP served as negative control. At 72 hours post infiltration, RCD1-GFP and YFP were immunoprecipitated with GFP-specific antibody and co-precipitating PPK-HA proteins were detected by αHA immunoblot. Immunoprecipitation of RCD1-GFP and YFP was confirmed by an αGFP immunoblot. Input samples were taken before immunoprecipitation and included on the immunoblots to test for equal expression and loading.

b. RCD1 co-localizes with PPKs in NBs in tobacco. RCD1-Venus was co-expressed with RFP or RFP-tagged PPKs in epidermal cells of *N. benthamiana* and the subnuclear localization was analyzed by confocal microscopy.
Scale bars indicate 10 µm.

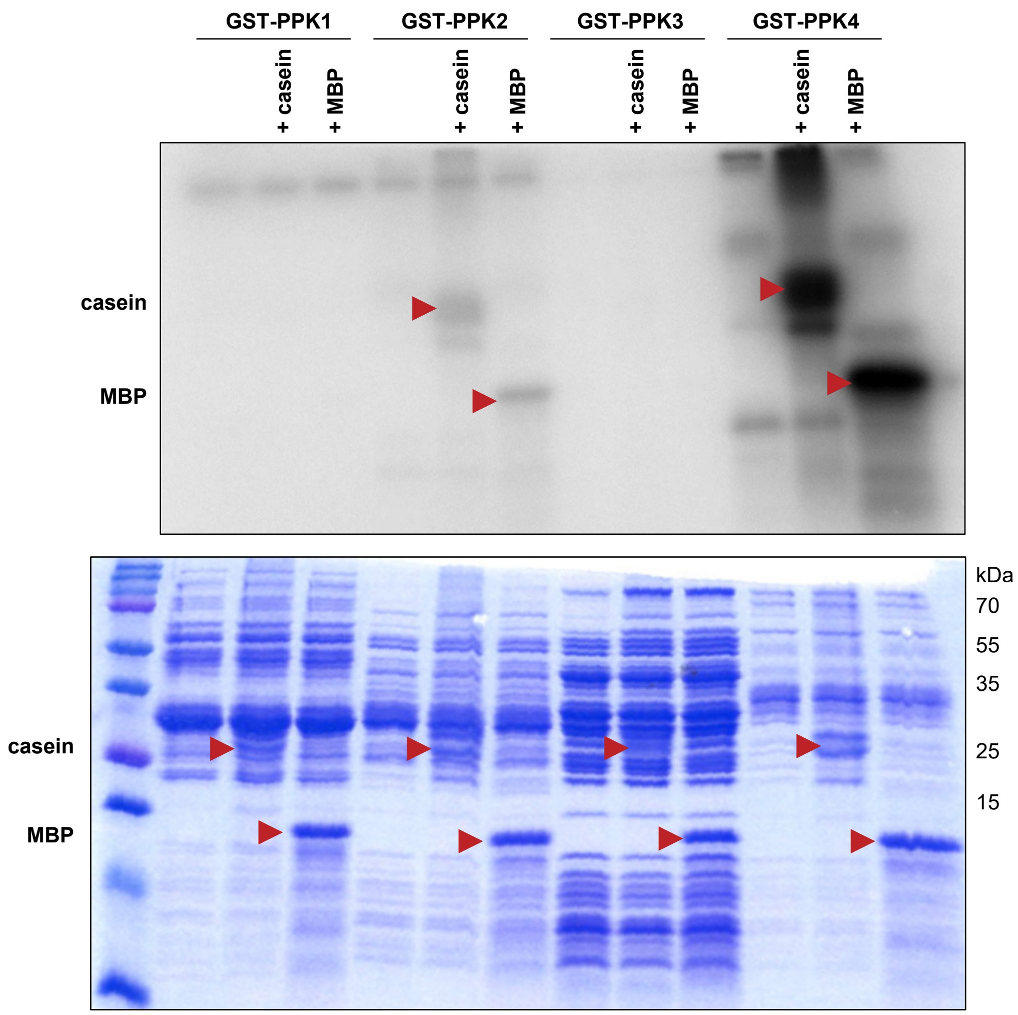

Supplementary figure 6. Recombinant PPK2 and PPK4 are active in *in vitro* kinase assays.

Recombinant GST-PPK1-4 were used together with generic substrates casein and myelin basic protein (MBP) in an *in vitro* kinase assay. Upper panel shows autoradiograph, lower panel shows the Coomassie-stained SDS-PAGE.

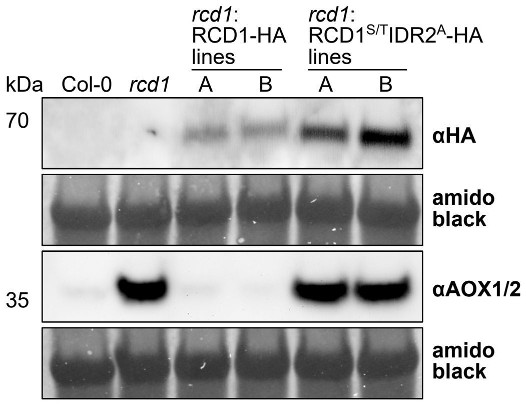

Supplementary figure 7. RCD1$^{S/T}$IDR2^{A}-HA protein accumulates at higher levels than RCD1-HA *in planta*.

In vivo abundance of RCD1$^{S/T}$IDR2^{A}-HA and of wild-type RCD1-HA variants was assessed in independent transgenic lines by immunoblot analysis with HA-specific antibody. The RCD1$^{S/T}$IDR2^{A}-HA variant did not fully complement *rcd1*-specific accumulation of alternative oxidases, as revealed by immunoblot with αAOX1/2 antibodies. Rubisco large subunit detected by amido black staining is shown as a control for equal protein loading..

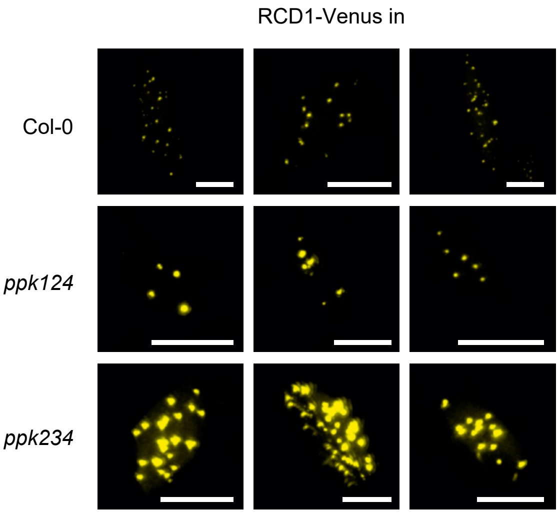

Supplementary figure 8. RCD1-Venus forms different NBs in Col-0 and *ppk* mutants.

Confocal images of RCD1-Venus NBs in mesophyll cells of 10-day-old T_2 Col-0, *ppk124* and *ppk234* seedlings. Images shown are maximum-intensity projections of z-stacks. Scale bars represent 10 µm.

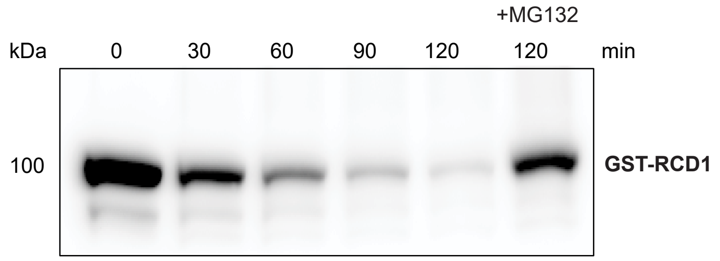

Supplementary figure 9. Proteasome mediated degradation of RCD1.

Recombinant GST-RCD1 was incubated with cell extracts for indicated time points in absence or presence of the proteasome inhibitor MG132 (+MG132). Protease inhibitors were added to the extracts before incubation with GST-RCD1. Degradation of GST-RCD1 is specifically prevented by MG132 as revealed by Western blot analysis with anti-GST antibody.

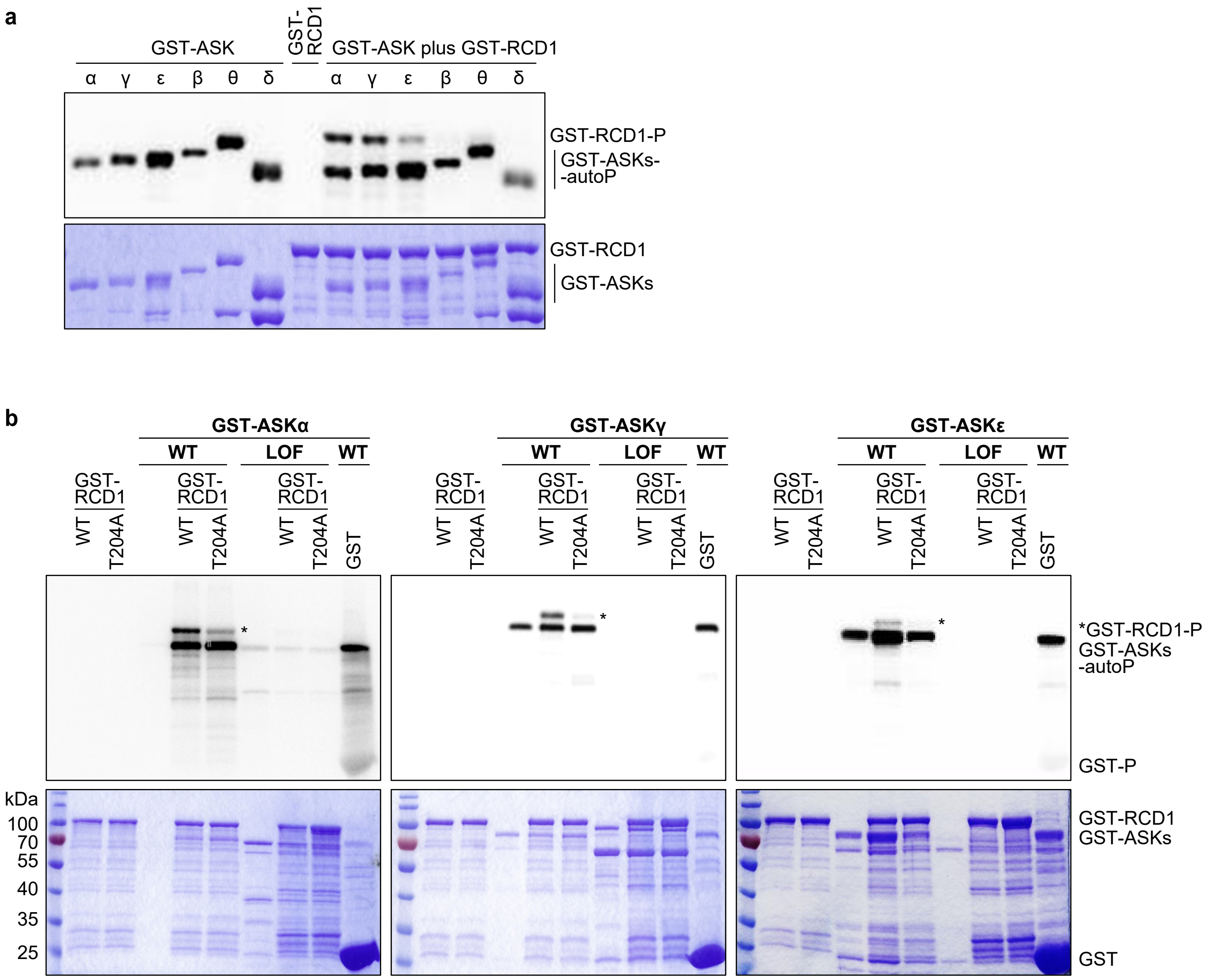

Supplementary figure 10. ASKα, ASKγ, and ASKε phosphorylate RCD1 *in vitro*.

a. Specificity of ASKα, ASKγ and ASKε towards RCD1. Recombinant ASK-GSTs were used together with recombinant GST-RCD1 protein in an *in vitro* kinase assay. P – phosphorylated protein; autoP – autophosphorylated protein.

b. Thr204 is the target for ASKs. GST-ASKα, GST-ASKγ and GST-ASKε were used with recombinant GST-RCD1 or GST-RCD1T204A in an *in vitro* kinase assay. LOF indicates loss-of-function constructsof ASKs.

Upper panels show autoradiographs, lower panels show the Coomassie-stained SDS-PAGE.

Supplementary figure 11. Uncropped and unedited immunoblot/ gel images

Figure 4c.

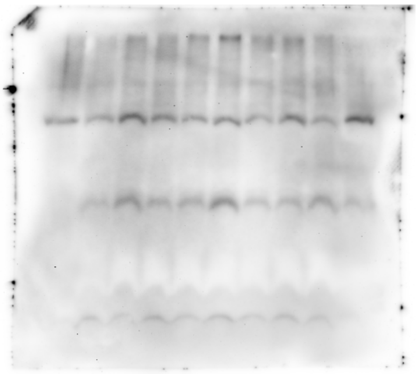

αRCD1

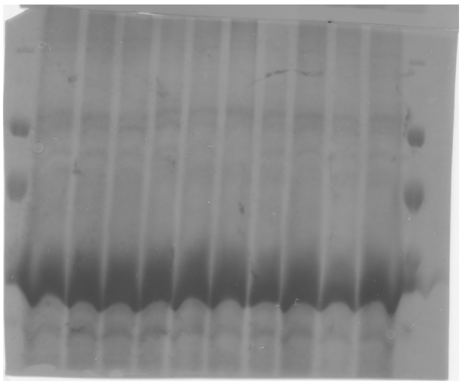

amido black

Supplementary figure 1d.

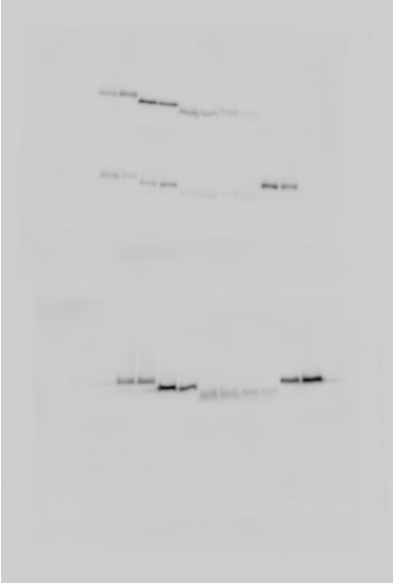

αHA

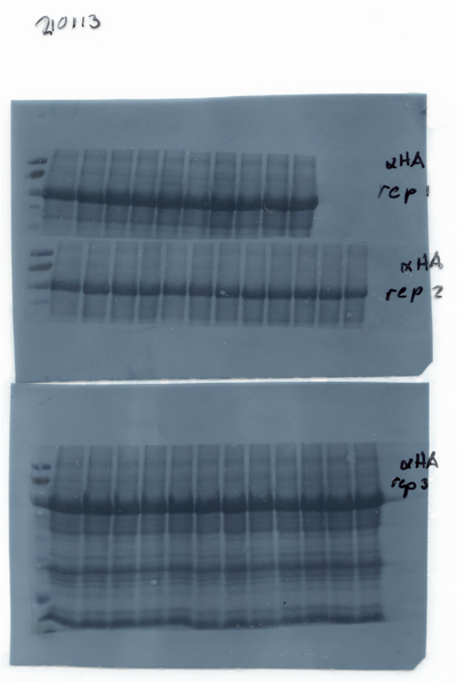

amido black

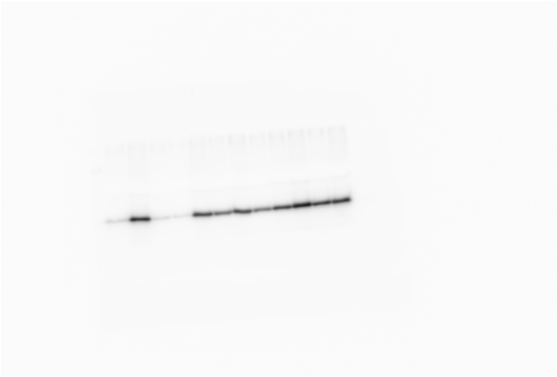

αAOX

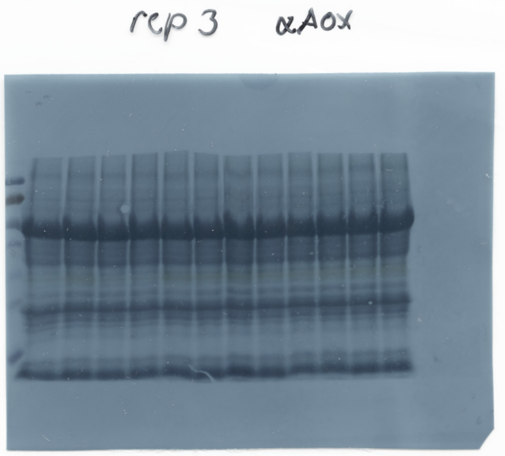

amido black

Supplementary figure 2b.

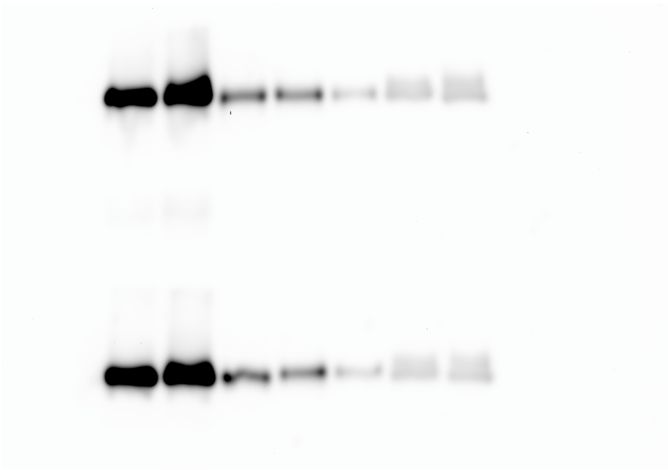

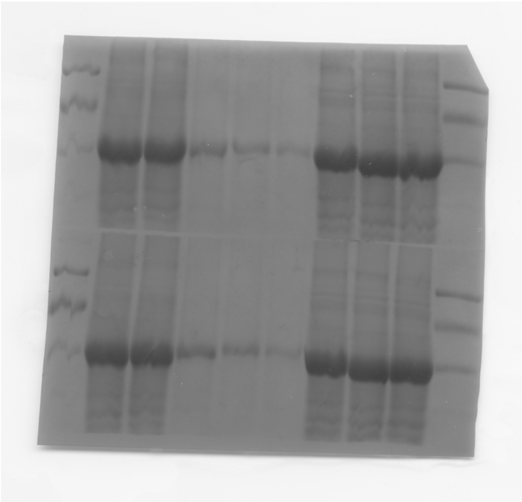

αHA amido black

Supplementary figure 3a.

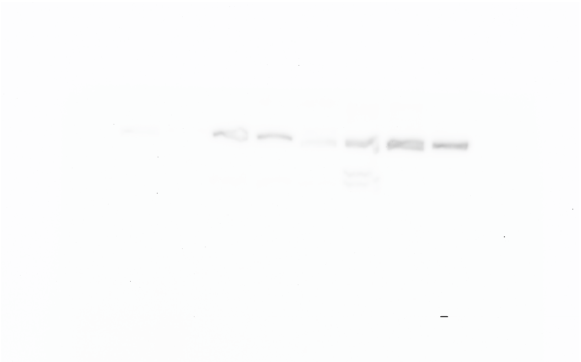

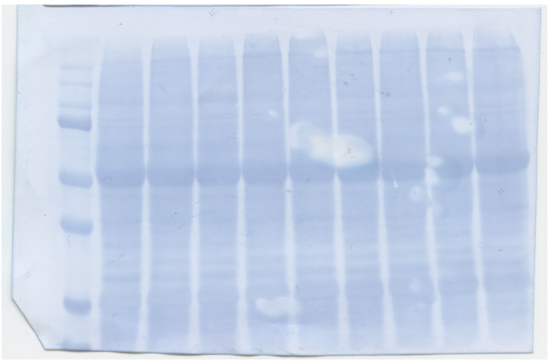

αHA amido black

Supplementary figure 3b.

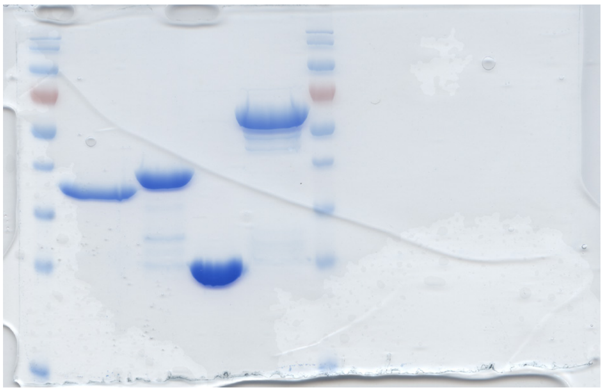

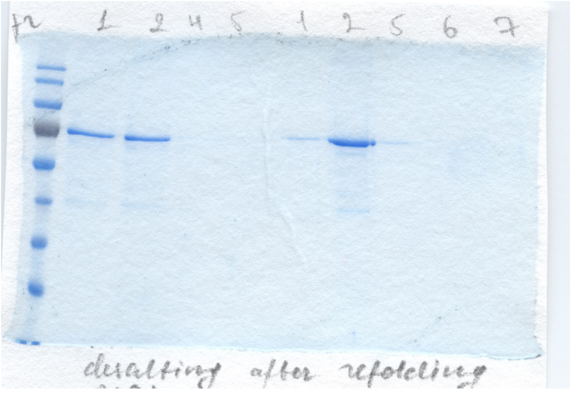

Supplementary figure 5a.

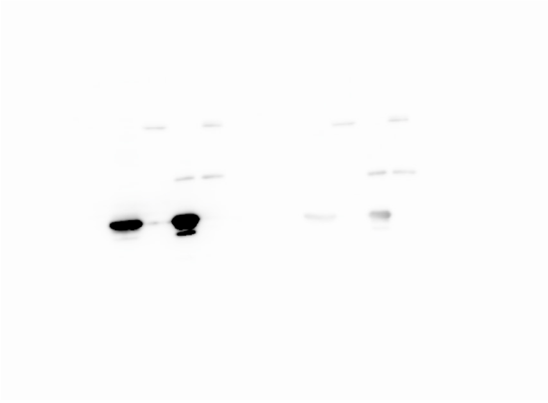

αGFP

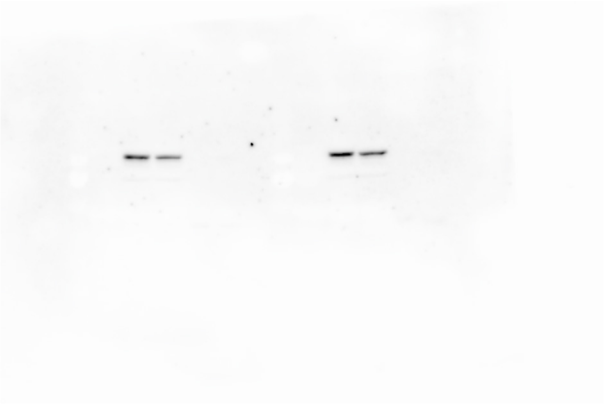

αHA

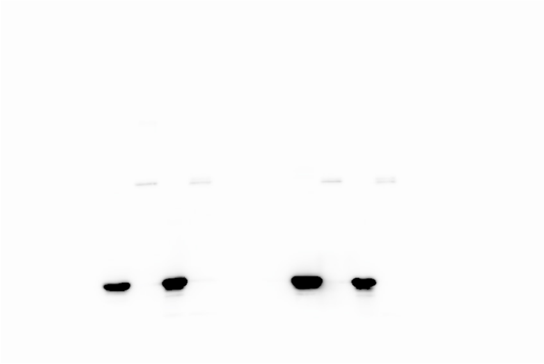

αGFP

αHA

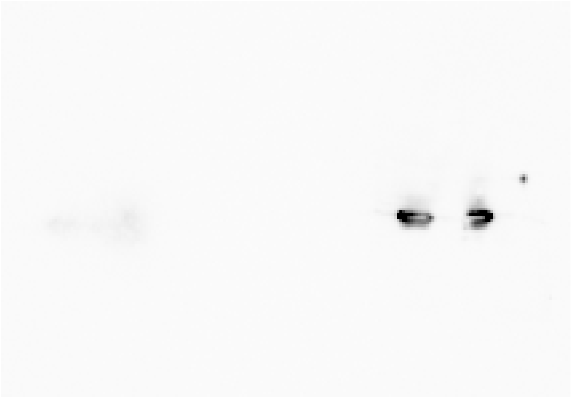

αGFP

αHA

Supplementary figure 7.

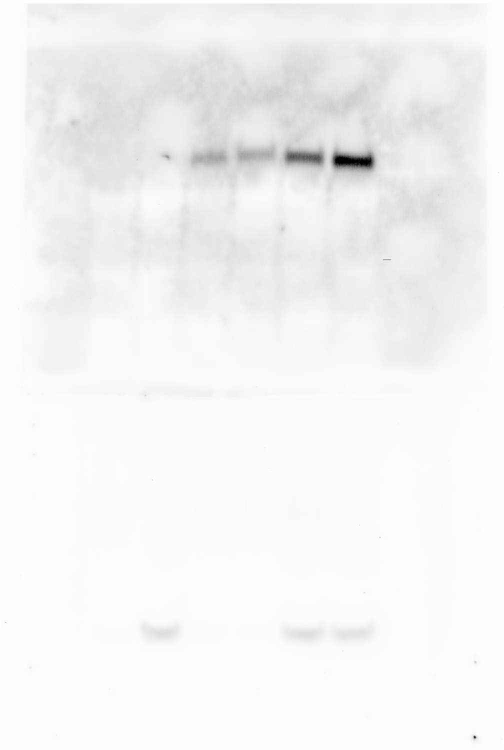

αHA

αAOX

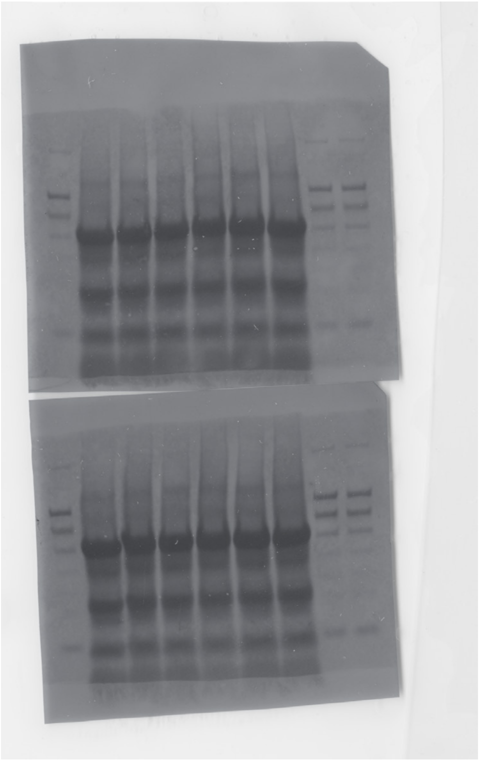

amido black

Supplementary figure 9.

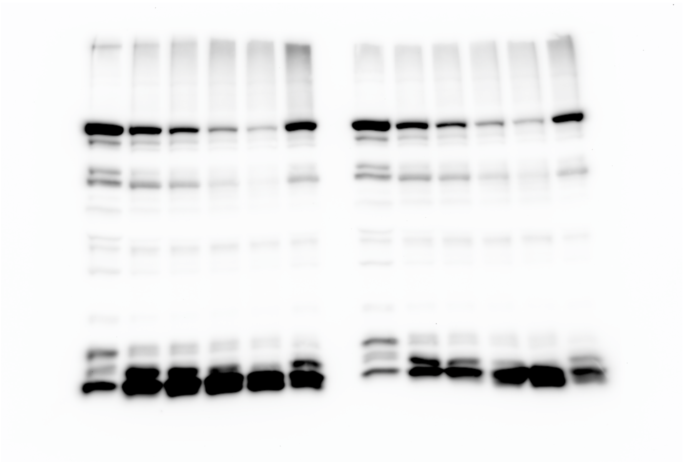

αGST
