# Supplementary material for: Poly(ADP-ribose)-binding protein RCD1 is a plant PARylation reader regulated by Photoregulatory Protein Kinases
Source: Commun Biol. 2023 Apr 19;6:429. doi: 10.1038/s42003-023-04794-2 (PMC10115779; doi:10.1038/s42003-023-04794-2)
Supplement: Supplementary file 3 — Description of Additional Supplementary Files [file 42003_2023_4794_MOESM3_ESM.pdf]

Description of Additional Supplementary Files

File name: Supplementary Data 1

Description: the source data and statistical tests behind the graphs in the paper.
